# Biosafety and biosecurity approaches to restrain/contain and counter SARS-CoV-2/COVID-19 pandemic: a rapid-review

**DOI:** 10.3906/biy-2005-63

**Published:** 2020-06-21

**Authors:** Tauseef AHMAD, Haroon, Kuldeep DHAMA, Khan SHARUN, Fazal Mehmood KHAN, Irfan AHMED, Ruchi TIWARI, Taha Hussien MUSA, Muhammad KHAN, D. Katterine BONILLA-ALDANA, Alfonso J. RODRIGUEZ-MORALES, Jin HUI

**Affiliations:** 1 Department of Epidemiology and Health Statistics, School of Public Health, Southeast University, Nanjing China; 2 Key Laboratory of Environmental Medicine Engineering, School of Public Health, Ministry of Education, Southeast University, Nanjing China; 3 College of Life Science, Northwest University, Xian China; 4 Division of Pathology, ICAR-Indian Veterinary Research Institute, Izatnagar, Bareilly, Uttar Pradesh India; 5 Division of Surgery, ICAR-Indian Veterinary Research Institute, Izatnagar, Bareilly, Uttar Pradesh India; 6 Key Laboratory of Special Pathogens and Biosafety, Centre for Emerging Infectious Diseases, Wuhan Institute of Virology, Chinese Academy of Sciences, Wuhan China; 7 Department of Physics, Government Postgraduate College, Mansehra, Khyber Pakhtunkhwa Islamic Republic of Pakistan; 8 Department of Veterinary Microbiology and Immunology, College of Veterinary Sciences, Deen Dayal Upadhayay Veterinary Science University and Cattle Research Institute, Mathura India; 9 Department of Genetics, Centre for Human Genetics, Hazara University Mansehra, Khyber Pakhtunkhwa Islamic Republic of Pakistan; 10 Semillero de Zoonosis, Grupo de Investigación BIOECOS, Fundación Universitaria Autónoma de las Américas, Sede Pereira, Pereira, Risaralda Colombia; 11 Public Health and Infection Research Group, Faculty of Health Sciences, Universidad Tecnologica de Pereira, Pereira Colombia; 12 Grupo de Investigacion Biomedicina, Faculty of Medicine, Fundacion Universitaria Autonoma de las Americas, Pereira, Risaralda Colombia

**Keywords:** COVID-19, SARS-CoV-2, 2019-nCoV, current scenario, biosafety & biosecurity

## Abstract

Emergence and reemergence of infectious diseases pose significant public health risks that are continuously haunting human civilization in the past several decades. Such emerging pathogens should be considered as a high threat to humans, animals, and environmental health. The year 2020 was welcomed by another significant virus from family *Coronaviridae* called severe acute respiratory syndrome coronavirus 2 (SARS-CoV-2) that caused the coronavirus disease 2019 (COVID-19). The disease was first reported in the city of Wuhan, Hubei province, China. Within a short time, this disease attained the status of the Public Health Emergency of International Concern. Presently, COVID-19 has spread to more than 150 countries, therefore, the World Health Organization (WHO) called it a pandemic. The Chinese government, along with WHO, other health agencies, and many nations, are monitoring the current situation closely to analyze the impact of SARS-CoV-2/COVID-19 on humans, animals, and environmental health. In the context of the current situation, biosafety and biosecurity measure that focus on One Health aspects of the disease outbreaks and the SARS-CoV-2 spread are of great importance to restrain this pathogen. Along with these efforts, standard precaution and control measures should also be taken at personal and community level to prevent the spreading of any contagion diseases, including COVID-19. Researchers are putting their very high efforts to develop suitable vaccines and therapeutics/drugs to combat COVID-19. This review aims to highlight the importance of biosafety, biosecurity, One Health approach, and focusing on recent developments and the ways forward to prevent and control COVID-19 in a useful way.

## 1. Introduction 

Emergence and reemergence of few potent and highly infectious diseases are posing significant public health risks that are haunting human civilization continuously, thereby posing a considerable threat to the lives of humans as well as animals all around the world. 

Historically, many deadly disease outbreaks have happened, few due to the mutations of the infectious pathogens, cross-species jumping of the microbes, and the majority of these were linked to zoonotic origins, and others may be due to evolving newer and novel and even highly pathogens (Table 1). The threats of such diseases can arise in any country and nations across the world. Previous studies have shown that 60% of the infectious diseases and 70% of emerging infections of humans have originated from zoonotic origins (WHO, 2020a). This might be due to unplanned urbanization, following integrating animal husbandry practices, increased contact with animals, and food-habits to consume different kinds of animals, poultry, and wildlife animal species (Mourya et al., 2019; Salata et al., 2020).

**Table 1:** Highly pathogenic viruses and dealing recommended biosafety levels

Viruses	Risk groups	Recommended laboratory
Ebola	4	BSL4
Nipah virus	4	BSL4
Hendra virus	4	BSL4
Chikungunya	3	BSL3
Japanese encephalitis	3	BSL3
SARS-CoV-2	3	BSL3
Hantavirus	3	BSL2
HPAI H5N1	3	BSL2
HIV	3	BSL2
MERS-CoV	3	BSL2
SARS-CoV	3	BSL2
West Nile	3	BSL2
Dengue	2	BSL2
Zika	2	BSL2

In recent times, the world has experienced deadly disease outbreaks such as severe acute respiratory syndrome-related coronavirus (SARS-CoV), Middle East respiratory syndrome-related coronavirus (MERS-CoV), bird flu, swine flu, Ebola, Zika, Nipah, and now facing the most recent threats of coronavirus disease 2019 (COVID-19), caused by severe acute respiratory syndrome coronavirus 2 (SARS-CoV-2) (Dhama et al., 2012; Munjal et al., 2017; Dhama et al., 2018; Singh et al., 2019; Dhama et al., 2020a; Malik et al., 2020; Rodriguez-Morales et al., 2020).

**Figure 1:**
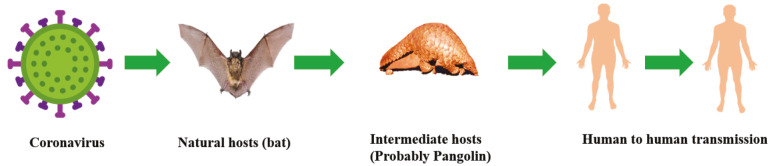
Transmission flow of COVID-19.

On December 31, 2019, 27 pneumonia cases of unknown aetiology had been reported by the Wuhan Municipal Health Commission, Hubei province, China. On January 7, 2020, the pathogen was confirmed as SARS-CoV-2, previously known as a novel coronavirus (2019-nCoV). Although other vector-borne diseases, zoonotic diseases, and snake bites are still matter of concern, COVID-19 became a pandemic, as declared on March 11, by the World Health Organization (WHO). Currently, COVID-19 has spread to more than 180 countries/regions (Coronavirus COVID-19 Global Cases, 2020). The disease has been reported to be initially transmitted from animals to humans, and then acquired efficient human to human spread in a rapid way (Ahmad et al., 2020a). The transmission cycle of SARS-CoV-2/COVID-19 is presented in Figure 1.

At present, there are not commercially marketed effective COVID-19 specific vaccines or antiviral drugs at our disposal. Hence, the current scenario warrants the enforcement of strict preventive and control strategies for minimizing the rapid spread of this fatal pathogen across the globe (Dhama et al., 2020b). Previously published literature suggests that the laboratory capacity, treatment, and diagnostic facilities were found to be inadequate in limited-resources countries (Heckert et al., 2011). One Health approach is needed for active investigation, prevention, and diagnosis of COVID-19 (Ahmad and Hui, 2020a). In the context of zoonotic diseases that affect both human and animal populations, it is a necessity that both human and veterinary experts must work together in cordial and cooperative networking modes. 

The source of the SARS-CoV-2 is very likely related to a wild animal, and there is a dire need for strict laws to limit wildlife markets to control the occurrences of new emerging zoonoses in the future (Wang et al., 2020). We know that biosecurity and biosafety are not the issues that can be addressed by only researches but need practical strategies and training in handling infectious pathogens and disease outbreaks situations across the global scenario (Dhama et al., 2013PRC, 2020). 

Therefore, looking into the future, we must continue to work together on an international level with high collaborative, cooperative, and networking efforts by adopting rapid means of communications and updates as well as supported with adequate health infrastructure, medicines, and intensive care units. A balanced and practical approach to global biosafety can be achieved only by recognizing the many viewpoints and approaches to biosecurity and biosafety procedures implemented effectively around the world to create a safer world of today and for the next generations to be safeguarded from any adverse health events of disastrous outbreaks such as SARS-CoV-2/COVID-19. For better understanding this purpose, we must shift our perception from national responsibilities to one of a global responsibility from the viewpoint of national, global, One Health approaches that may encourage all the entire community to share their experiences and bring safety at present and future. Researchers and health agencies across the globe are making continuous high efforts to check and combat the spread of this virus, along with developing effective vaccines and therapeutics/drugs (Ahmad et al., 2020b). 

The present review describes in brief some salient features and aspects of biosafety, biosecurity, and One Health approaches, and their usefulness, potent roles, and applications to restrain/contain and counter COVID-19 disease outbreaks, and check the further spread of this emerging disease.

## 2. COVID-19 as a global threat 

SARS-CoV-2/COVID-19 is a severe threat to public health as a pandemic. Also, factors such as high human migration, air traffic, and trade between China and other countries pose risks. Some of the countries in Africa are under high risk of introduction and spreading of the COVID-19 (Nkengasong, 2020; Nkengasong and Mankoula, 2020). Primarily the travellers from China have imported COVID-19 cases to other countries such as the United States of America (USA) (Jernigan, 2020). The first case of COVID-19 outside mainland China was reported in Thailand (Today, 2020). In the Africa continent, the first case was reported in Egypt (WHO, 2020b). The COVID-19 outbreaks have created extra pressure on the world health systems to invest heavily in diagnosis, treatment, and for controlling the spread of disease, as well as to check the zoonotic risks. On the other side, the oil demand at a global level has been struck by COVID-19 outbreaks. Also, trade, business, movement, tourism, and other daily activities are slowdown and affecting companies around the world, including Nissan and Apple, and considered to be the most significant danger to the global economy (WEF, 2020). In low economic countries, the laboratory capacity is insufficient, and these facilities are poorly maintained (The PLoS Medicine Editors, 2007; Kruk, 2008; Wertheim et al., 2010; WHA, 2020). Therefore, to diagnose and treat such highly infectious pathogens such as (SARS-CoV-2/COVID-19), which can cause deadly diseases in humans, need well-established biosafety and biosecurity laboratories as well as the adoption of appropriate One Health approaches. 

## 3. Laboratory biosafety

The WHO describes the laboratory biosafety as the word used to define technologies, containment principles, and practices that are applied to stop unintentional exposure to toxins and pathogens or their accidental release. Furthermore, the laboratory biosecurity is described as the institutional and personal security procedures planned to inhibit the loss, misuse, diversion, theft, and intentional release of toxins and pathogens (WHO, 2020c). 

### 3.1 Laboratory biosecurity

Laboratory biosecurity is more than merely a kind of physical security. It also contains personnel-management, material accountability and control, transportation security, information security, and program management (Salerno et al., 2007). A biosafety laboratory (BSL) comprises a set of safety measures mandatory for handling hazardous biological agents in a secure, safe, and enclosed control setting (Table 1). Mainly, a BSL contains primary protective barrier means safety- equipment, and a secondary protective barrier means for safety facilities. There are 4 protection levels of biosafety laboratory designed to handle suitable pathogens in vitro named as BSL-1, BSL-2, BSL-3, and BSL-4 (Table 1); contrastingly, facilities designed to handle suitable pathogens in in-vivo experiments are named as animal biosafety laboratory (ABSL-1, ABSL-2, ABSL-3, and ABSL-4 (Zhiming, 2019). These biosafety levels based on the construction, design structures, equipment, containment facilities, standard practices, and operational techniques are warranted while working with various infectious and deadly agents of different risk groups. 

BSL-1: It needs no special restraint equipment and is used for well-characterized agents not identified to cause infections in healthy humans and that have an insignificant possible threat to the environment. 

BSL-2: BSL-2 is applicable for dealing pathogenic agents related to human infections that are less pathogenic and for which preventive and therapeutic mediations are available. 

BSL-3: BSL-3 is designed for dealing with pathogenic agents that can cause severe infections and deadly diseases in humans. This level represents those pathogenic agents who are having high individual risks but low community risks. For this group, pathogenic agents, preventive, and therapeutic mediations may be available. To work in BSL-3, scientists and researchers need to wear the proper protective clothing and equipment in negative pressure rooms. 

BSL-4: BSL-4 is the extreme control laboratory that is designed and constructed for the dealing of highly pathogenic and dangerous biological agents that can cause severe and deadly infections in humans. This level represents pathogenic agents who are having high individual and community risks. Mostly for this group, no preventative or therapeutic interventions are available. For the researchers who are working in BSL-4 laboratory it is mandatory to wear positive pressure personnel suits (WHO, 2020c; CSNC, 2020). 

## 4. Biological threats to One Health

Infectious disease agents and toxins constitute noteworthy threats to the economy, biodiversity, food security, food safety, and public health. Therefore, integrating veterinary and human public health surveillance and monitoring efforts are essential to control such deadly infectious agents which could pose pandemic threats and put billions of human populations at high risks for their survival (Rabinowitz et al., 2006). The WHO recognizes zoonoses as emerging threats and defines them as phenomena which have an increasing trend and tendency of expansions in different geographical regions, hosts, or vector’s range. As the global human population increases, the probability of zoonotic pathogen transmissions from animals to humans increases with anthropological pressures on animals and the environment (Singh, 2009; Bonfoh et al., 2010). The problem of not adopting adequate biosecurity measures is most acute in animal husbandry practices including of livestock and poultry production, especially the threats posed from transboundary animal diseases (TAD), relevant to endemic diseases such as Peste des petits ruminants (PPR), bluetongue, swine fever, infectious pustular vulvovaginitis (IPV), caprine encephalitis (CAE), maedi-visna, equine piroplasmosis, rhinopneumonitis of the equine, bovine viral diarrhea (BVD), bovine immunodeficiency (BIV), and few others. Such pathogens are responsible for causing enormous economic losses, and therefore need to have strategies for biosecurity in place to be strictly executed. Therefore, efforts to prevent pandemic diseases, categorized into external biosafety and internal biosecurity, need to be aimed at preventing the spread of infection (Singh, 2009; FAO, 2008). 

With more livestock and animal products moving longer distances in shorter periods and rising concentration of food production to fewer countries and production companies, the world is becoming increasingly vulnerable to the adverse effects of animal diseases. Endemic animal diseases in some of the world’s poorest countries are a constant threat to health and agriculture, hampering economic and social development, and reducing food availability (WOAH, 2015). 

**Figure 2:**
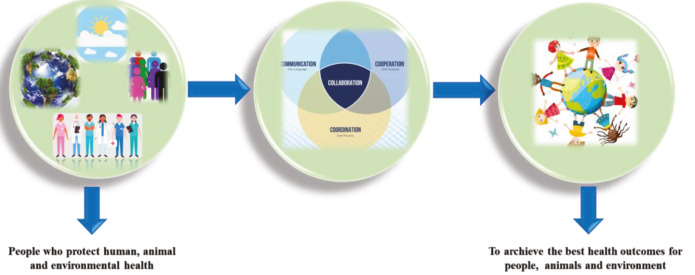
One Health approach, modified from Ahmad and Hui (2020).

The Centers for Disease Control and Prevention (CDC) USA, planned to combat biological terrorism attacks and develops guidelines for investigating and assessing suspected human clusters and animal diseases or injury, and triggers for notifying law enforcement of prevention, control and restricting possible biological or chemical terrorism incidents and threats (Rabinowitz et al., 2006). Similarly, biological terrorism has increased the number of sick or dead animals, and biological warfare agents can intoxicate these animal disease outbreaks triggered by bioterrorism agents to a wide variety of hosts (Rabinowitz et al., 2006). In the context of the current epidemic of COVID-19, the One Health approach should be of great importance (Ahmad and Hui, 2020b). A diagrammatic representation of the One Health approach is presented in Figure 2. 

## 5. Highly pathogenic emerging viruses

Emerging viruses are those viruses that have caused disease outbreaks in the past 2 decades of the 21st century. These emerging viruses are also defined as those viruses which have posed probable threats of disease outbreaks in the coming years. Emerging viruses consist of those viruses that are detected in people as a new or the ones that may have been existing earlier but are now increasing at a higher rate in the global population (Morens et al., 2004; Howard and Fletcher, 2012). Some of the examples of highly emerging viruses are avian influenza (HPAI) virus of subtype H5N1, Ebola virus, Middle East respiratory-syndrome coronavirus (MERS-CoV), severe acute respiratory syndrome (SARS), Chikungunya virus, Zika virus, Japanese encephalitis virus, West Nile virus, Hantavirus, Hendra virus, and Nipah virus. These highly pathogenic viruses cause diseases which are highly threatening animal and human health (Morens et al., 2004; Jones et al., 2008). In the context of the above statement, SARS-CoV-2 is also a deadly pathogenic virus that caused a considerable risk to public health and accounted for very high economic losses in a short period of 2 months. 

### 5.1. Biosecurity for dealing pathogenic emerging viruses

For working on pathogenic microorganisms, it is essential to have proper biosafety and biosecurity laboratories. The purpose of biosecurity is to prevent the spreading out of the emerging pathogenic viruses from the safety laboratory into the outside environment. In the biosecurity laboratory, there should be specific professional working groups, which may include science directors, principal investigators, professional laboratory staff, administrators, facility maintenance personnel, laboratory safety officers, law enforcement agencies, and security staff (CDCP, 2009). The biosecurity laboratory contains a system of a multidimensional approach, which includes physical building security, biohazard material accountability and control, personnel security, information security, and transport security (Gaudioso and Zemlo, 2007). Table 2 is presented with a list of highly pathogenic viruses including SARS-CoV-2, and dealing with recommended laboratories/facilities. 

**Table 2:** Animal’s impact on human health around the globe: enlisted diseases are vector-borne diseases, zoonotic diseases, and snake bites.

	Death/year	People affected/year
Vector-borne diseases	1.0 million	1.0 billion
Malaria	0.6 million	500 million
Dengue	20,000	50–200 million
Zoonotic diseases		
HIV	1.5 million	35 million
Gastrointestinal	1.23 million	1.7 million
Tuberculosis (zoonotic)	0.1 million	0.55 million
Rabies	70,000	70,000
Snake bites	20,000 to 11,000	0.4 million–2.4 million
Worker		
Commercial fishing	24,000	

## 6. Epidemiology, surveillance and monitoring–current positions and focusing a need to be enhanced 

The concept of public health surveillance is widely used in modern days with its contribution to provide data and information, assess, characterize and study the distribution of diseases and health adverse events, identification of emerging health conditions, prioritize public health actions, and monitor the control and prevention activities. In recent years, scientific advances in public health surveillance, rapidly evolving technologies, and changing health care requirements are of great importance. However, researchers and public health professionals continue to pursue developing new methods to evaluate and improve the ability of public health and disease surveillance systems to support new uses of data. Researchers are interested in the early recognition and management of chronic diseases (Groseclose and Buckeridge, 2017; Makam et al., 2013). In the context to support early detection of public health events and diseases, the syndromic surveillance system was introduced to alert at very low thresholds. Also, the practitioners and public health professionals have tried to improve the accuracy of syndromic surveillance systems by comparing results from different surveillance systems and modifying statistical outbreak detection algorithms (Groseclose and Buckeridge, 2017; Cadieux et al., 2012). Disease and public health surveillance are critical for public health, to timely recognize and collect data of public health events and outbreaks efficiently and effectively. In the context of the current epidemics of COVID-19, the epidemiological approaches, preventive strategies, extending disease surveillance at national and international level is essential to contain this disease that has created havoc and posed threats to the survival of humankind. 

## 7. Role of bats and other animals in SARS-CoV-2 

The recently emerged COVID-19 has been linked with a local live animal and seafood market in China (Perlman, 2020). It has been identified that apart from other various coronaviruses (CoV) hosts, bats and birds are the ideal hosts for CoVs. *Alphacoronavirus *and* Betacoronavirus *were identified in bat CoVs, while *Gammacoronavirus *and *Deltacoronavirus* dominate in bird CoVs (Liu et al., 2016; Woo et al., 2012). Likewise, in the phylogenetic analysis, it has been shown that MERS-CoV is closely linked to *Betacoronavirus* found in bats of Europe and Africa. Similarly, other examples of bat viruses that are found in humans and agricultural animals as their secondary host are filoviruses (Marburg virus and Ebola virus), porcine epidemic diarrhea (PEDV) virus, and henipaviruse (Nipah and Hendra viruses) (Moratelli and Calisher, 2015). 

However, since the first outbreak of COVID-19, intense research studies have been done to identify the source of origin of its causative agent (SARS-CoV-2). Paraskevis and colleagues, by using phylogenetic analysis, determined that COVID-19 is closely related to the Bat CoV RaTG13 sequence throughout its genome with a similarity index of 96.3%. However, especially in the middle region of nucleotide, almost half of the spike region, both COVID-19, and RaTG13, grouped in the form of separate distant lineage (sarbecovirus branch) (Paraskevis et al., 2020). Nevertheless, as bat population constitute a genetically diverse group and studies have shown that one species of a bat may not represent the other species of bats. However, the myth of the exact mechanism of the spread of the SARS-CoV2 virus is elusive and unclear. Therefore, to gain a deeper understanding of the microbe coevolution of a bat, its immune system and ecological interaction need to be unrevealed. Collaboration between state-of-the-art laboratories across the globe should make efforts to develop cell lines from particular tissues and reagents of a bat and initiate a setup of bat colonies in a laboratory for better understandings of microbe-host interaction in these ecological mammals. 

Both the initial origin and the primary route of transmission in the case of the COVID-19 outbreak are both still unknown. Since the first cases were reported from the live-markets in Wuhan, wild animals sold in the market might have played a possible role in the origin of the SARS-CoV-2 virus. Taking into consideration the possible involvement of an intermediate host, a temporary ban was imposed on the wildlife trade in China (Harypursat and Chen, 2020; Mallapaty, 2020). COVID-19 outbreak can also occur, employing the primary food-borne route, which is then further transmitted by the respiratory way among human beings (Jalava, 2020). The predecessor of SARS-CoV-2 was found to have originated from bats. Investigations conducted in the SARS and MERS CoVs outbreaks identified civets and dromedary camels as their intermediate hosts that played a significant role in the disease transmission to humans (Park et al., 2020). 

Advanced genomic analysis has pointed out that SARS-CoV-2 is closely related to the bat CoV that was previously isolated from horseshoe bat, the same species of bat that was found to harbor SARS-related CoVs by acting as its maintenance hosts. Hence, we cannot rule out the possibility of SARS-CoV-2 emergence as a sequel of sequential recombination occurring among the precursors of SARS-related coronaviruses. This is the reason for the extensive search of an intermediate animal host in COVID-19 that is responsible for the zoonotic spillover from bats to humans (Murdoch and French, 2020). It is very much essential to make a big decision regarding the trade of wild animals in China to prevent the possibility of future outbreaks due to zoonotic spillover. However, a total ban on the wild animal trade is not a practical solution as it will affect the livelihood of a substantial proportion of the Chinese population. Hence, such a move will only have a negative effect since it will shift the trade of wild animals to the black market (Mallapaty, 2020).

## 8. Diagnosis of COVID-19 

According to the new policy of the Food and Drug Administration (FDA), it can issue an u1d53 (EUA) for specific laboratories seeking to develop diagnostic tests to develop a rapid diagnostic testing method and facility to detect SARS-CoV-2. On February 4, 2020, the Secretary of Health and Human Services (HHS) determined that COVID-19 is a public health emergency, and the current circumstances exist for justifying the authorization of emergency use of in vitro diagnostics (FDA, 2020). Initially, the SARS-CoV-2 was isolated from a clinical sample, and within weeks many sensitive and reliable diagnostic tools were developed and deployed across the country. Furthermore, China has shared the information on viral sequences, PCR primers, and probes with the international community and WHO to facilitate further investigations and research on COVID-19. The virus sequence was uploaded to GISAID Database. On February 23, 2020, the National Medical Product Administration (NMPA), China has approved 10 detection kits used for COVID-19 diagnosis including virus sequencing product (01), isothermal amplification kit (01), colloidal gold antibody detection kits (02), and RT-PCR kits (06). The rapid serological diagnostic kit is being developed but not widely used (Report, 2020). Detection of SARS-CoV-2 from respiratory secretions Quantitative Real-time RT-PCR (RT-qPCR) assay has routinely been used. Besides, more than 7 types of diagnostic kits have been developed for the detection of the nucleic acid of SARS-CoV-2. In the context of the current situation in China, especially in Hubei province for management of the outbreak, a combination of RT-qPCR or NGS and clinical criteria may be more critical.

Studies have found that asymptomatic patients secrete similar viral load when compared to symptomatic patients. This is critical since the transmission capacity of minimally symptomatic or asymptomatic COVID-19 patients is very high and equally comparable to symptomatic patients indicating that the transmission can occur early in the course of infection (Zou et al., 2020). Diagnosis of COVID-19 is based on the detection of nucleic acids from the samples such as throat swabs, sputum, saliva, lower respiratory tract secretions, stool, blood, tears, and conjunctival secretions (Shen et al., 2020; To et al., 2020; Xia et al., 2020). In a recent study, anal swabs were found to give more positive results in the later stages of the infection compared to the oral swabs (Zhang et al., 2020). Hence, the clinicians and lab workers should take extra precautions while handling test samples even in the case of negative oral swab test results due to the possible fecal-oral transmission in COVID-19. 

Compared to the respiratory samples, the viral loads in stool samples were found to be less. Still, precautionary measures need to be followed while handling the stool samples since the possibility of disease transmission remains the same (Pan et al., 2020). The live virus was also detected in the self-collected saliva of patients infected with COVID-19; hence saliva can be considered as an ideal sample due to the noninvasive nature of sample collection technique for the diagnosis of COVID-19 infection in suspected individuals (To et al., 2020). The presence of SARS-CoV was also identified in the tears and conjunctival secretions of COVID-19 infected patients. Among the study population, only one sample that is obtained from COVID-19 patients with conjunctivitis gave positive RT-PCR results (Xia et al., 2020). This indicates the possibility of disease transmission through tears and conjunctival secretions in COVID-19 patients with conjunctivitis. The patients exhibiting clinical signs of COVID-19 may not test positive in nucleic acid detection tests at every stage of the disease. Both the positive and negative test result of patients exhibit almost similar clinical symptoms, thereby increasing the possibility of misdiagnosis or missed diagnosis. The only difference that observed in patients with positive test result is that they tend to have dyspnea (Li et al., 2020). 

Key factors: community containment, social distancing, isolation, and quarantine to prevent the spread and transmission of COVID-19.

Isolation: Isolation is the separation of sick peoples from healthy peoples.

Quarantine: Quarantine is the movement restriction of peoples, often with fever investigation, of contacts when it is not evident whether they have been infected and not yet symptomatic. 

Community containment: Community containment comprises procedures that range from increasing social distancing to wide-ranging community quarantine.

The COVID-19/SARS-CoV-2 is believed to be primarily transferred by respiratory droplets with similar generation time and incubation time as SARS coronavirus (SARS-CoV) (Zhu et al., 2019; Wilson and Chen, 2020). SARS-CoV was alarming at that time, maybe even more terrifying compared to COVID-2019, given its much more common progression of sickness and death. Nevertheless, the precise pieces of evidence were capable of absolutely interrupting human to human transmission, to end the epidemic, and therefore, SARS-CoV is now eliminated. In the absence of antivirals and vaccines, this significant success was only possible because of the strict implementation of appropriate and timely prevention and control strategies along with suitable public health measures and awareness programs (Wilder-Smith and Freedman, 2020). 

China again faced with the same situation of a coronavirus outbreak with a closely related virus-like SARS-CoV for which currently no specific vaccines and medicines exist. Still, time is needed to implement classical public health measures to control the epidemic of COVID-19, which causes a potentially life-threatening respiratory disease. The primary goal of public health procedures is to separate people in order to minimize the risks of the spread and prevent person to person transmission of this virus. The measures by which we can control and reduce the chances of transmission and check to spread of COVID-19 are community containment, social distancing, isolation, and quarantine. The Chinese government has implemented all these measurements for the effective control of COVID-19. In order to offer quick responses to the outbreaks of COVD-19 and immediately implement all the necessary measures within a week to minimize the risks of transmission and control the spread of this viral infection, following case detection, immediate isolation and contact tracing are implemented and medical follow-ups and intensive care are provided to all the people affected (Wilder-Smith and Freedman, 2020).

## 9. Public recommendations for avoiding the unprotected contact with both farm and wild animals

In Wuhan, China, the newly discovered COVID-19 disease outbreaks triggered and alarmed a health official around the world to start issuing advisories that provided necessary steps that people could take to help reduce the risks of this CoV infection. Many of the suggested preventive precautions require some simple common-sense, preventive health methods which can be best practiced at all times, such as washing hands with soap/detergent, water and disinfectant, covering the mouth during coughing or sneezing, avoiding close contact with individuals experiencing clinical symptoms and following appropriate kitchen practices while cooking animal-based food items thoroughly (Khan and Naushad, 2020; Moritsugu and Miller, 2020). 

At a seafood and meat market in Wuhan, China, where live animals were slaughtered and sold as food, the new virus appears to have jumped from wildlife to humans. That was a familiar story of the SARS-CoV outbreak, which was also caused by a coronavirus started eating a catlike animal called then palm civet in China. The MERS-CoV epidemic began and was transmitted from dromedary camels into humans in the Middle East. Conservationists see a lesson on public health in the spread of yet another coronavirus. The WHO issued a coronavirus-related graphics of animals warned against unprotected sex with wild animals. It is of great importance to avoid unsafe interaction with wild life or farm animals (vanUhm, 2016; Mikkelson, 2020). 

A common way in which people can get infected with germs that can cause zoonotic diseases is the close connection between humans and animals, including direct contact, encountering the saliva, blood, urine, mucous, feces (waste), or other body fluids. Examples include animals being petted or handled, and bites, or scratches: indirect contact where the animals are living and moving, or artefacts or surfaces contaminated with germs. Examples also include water from the aquarium tank, pet habitats, chicken coops, barns, trees, and soil, and pet food and water dishes. While some people eat or drink anything unhealthy, like unpasteurized (raw) milk, undercooked meat or eggs, or fresh fruits and vegetables tainted with infected animal feces (CDCP, 2020). Contaminated food can cause disease in both humans and animals, including poultry. Everyone, including healthy people, can get sick from the zoonotic disease. Some individuals, however, are at higher risk as compared to others and need to adopt appropriate necessary steps to protect themselves or their family members from zoonotic efforts. Some persons are more likely to get sick and may even die from infection of certain diseases than others, such as children younger than 5 years, individuals older than 65 years, individuals with weak, compromised immune systems (immunosuppressed persons), and pregnant women in particular.

## 10. Live-animal markets to be avoided which are posing novel zoonotic pathogens threats, public health risks during an outbreak

A pathogenic microorganism (bacteria, virus, parasite, or fungus) that is carried by animals and transferred to humans is called zoonosis or zoonotic disease (Brucker, 2020). The disease presents mild symptoms of respiratory infection, the same as occurs in the common cold. Still, sometimes this zoonosis or zoonotic disease can be so fatal and lethal that it could cause a pandemic. The significant sources implicated in zoonosis are animal vector-borne diseases, zoonotic diseases, snake bites, and tick (Scanes, 2020). According to CDC, the primary food sources that cause food-borne diseases and result in hospitalization are associated with meat, dairy, poultry, beef, and pork consumption, with proportions of 45.5%, 11.5%, 5.4%, and 5.1%, respectively (Painter, 2013).

Similarly, molluscs (oysters and mussels) are filter feeders, and pathogens are accumulated in these, and hence there is a risk of foodborne diseases (Grodzki et al., 2012). Noroviruses were detected in oysters in Europe, likewise as in cattle, and pigs are identified rotaviruses, and these animals are considered as the primary source of zoonotic infections (Midgley et al., 2012). According to the previous research findings and related to the current scenario of COVID-19, it has been recommended by Chinese health authorities to avoid visits to live-animal markets. In this regard, the unknown current source of the epidemic might be animals or birds until or unless the cause of disease transmission is found.

## 11. Tips, guidelines, and policy pertinent to travellers, general public/and prevention of infectious disease

Chinese health officials have confirmed thousands of COVID-19 affected cases in China, with the virus widely spreading in many parts of the country from person to person. COVID-19 cases, most of which are associated with travelling from Wuhan, are also being reported in a growing number of international locations (NCIRD, 2020). There is currently no vaccine or drug available for COVID-19. In such a scenario, the community-based interventions such as school dismissals, event cancellations, social distancing, and the creation of staff plan to work remotely can help slow down the spread of COVID-19. Individuals can take daily preventive measures, such as frequent hand washing, staying at home when they are sick and coughing and sneezing (CDC, 2020; Wu and McGoogan, 2020). Such infections may lead to severe symptoms in individuals having a weak immune system, older aged people, and those suffering from long-term illnesses such as diabetes, cancer, and chronic lung diseases (MacLaren et al., 2020; GOV.UK, 2020). Self-observation means that people should remain alert to subjective fever, cough, or difficulty in breathing so that they can timely contact the nearest health care providers or their local health departments immediately to determine whether a medical assessment is needed.

Therefore, the patient should be monitored for fever by taking temperatures twice a day and need to remain alert to coughing or difficulty in breathing. The State or local public health authority shall be responsible for assessing exposed persons for the presence of fever, cough, or difficulty in breathing and high-risk exposures, as recommended by CDC for this communication to occur at least once in a day (CDC, 2020; Habibi et al., 2020). The COVID-19 disease also spreads from person to person. Therefore the isolation and quarantine of a person or a group of people known or reasonably believed to be infected with communicable disease and potentially infectious from those who are not infected to prevent the spread of infectious disease not yet symptomatic are warranted to check its spread (Brooks et al., 2020; Rasmussen et al., 2020). Controlled travel includes excluding long-distance commercial aircraft, ships, trains, and buses, and continuous monitoring of travel should be coordinated with public health authorities. Moreover, avoid setting up close to congregate settings (public places), such as shopping malls, movie theatres, arenas, offices, schools, and classrooms (CDC, 2020).

## 12. Prevention and control of COVID-19 

**Figure 3:**
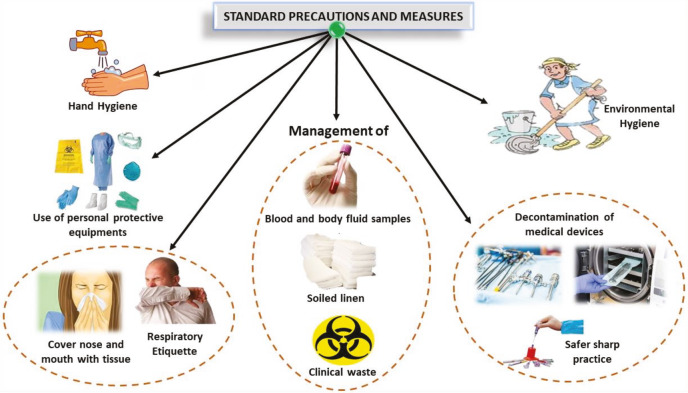
Standard precautions to prevent the spread of SARS-CoV-2/COVID-19.

It is of great importance to stop further spreading of SARS-CoV-2/COVID-19, for this purpose the standard precautionary measurements should be followed, as presented in Figure 3. The COVID-19 has kept alert the entire health institute across China and also at a global level as it can spread from person to person. Therefore, massive control strategies and appropriate responses and preparedness plans have been implemented (Daszak et al., 2020). Since the outbreak occurred, there is no vaccine or specific antiviral treatment to prevent COVID-19. However, prevention and control strategies received at the community level have become helpful by reducing the transmission routes of the disease. The SARS-CoV-2 nucleic acid is used as a detected method for specimens, including nasopharyngeal swabs, sputum, lower respiratory tract secretions, blood, and stool (Zhou et al., 2020).

Prevention and control of the transmission of COVID-19 is a crucial issue for health workers for proper management and checking the outbreaks of this emerging disease. The primary mode of communication of COVID-19 infection is through direct contact from person to person or exposure to infectious persons through the respiratory droplets or fomites. Therefore, it is recommended to maintain necessary good personal and environmental hygiene practices and to implement stringent avoidance of the contact and droplet precautions among health care workers, which can reduce the cumulative incidences of the COVID-19. To prevent community transmission, contact tracing, quarantine/isolation of close contacts, and public education are the essential measures that should be carried out, besides implementing droplet precautions and contact precautions seem adequate to reduce the risks of SARS-CoV-2 virus infection. 

Hence it is recommended to avoid close contact with affected people/infected individuals, not to touch eyes, nose, and mouth frequently, cover-up cough, or sneeze with a tissue, after that, dispose of the used tissue in the dustbin. Added to this, appropriate cleaning and disinfection procedures should be followed frequently for touched/contaminated objects, fomites, and surfaces by employing standard and regular household cleaning and disinfectant sprays or wipes, besides the recommendations of WHO, China CDC for COVID-19 outbreak should also be followed. Furthermore, exposure to patients with mild SARS-CoV infection, airborne precautions (hand hygiene, gown, gloves, N95 masks, and eye protection) should be implemented if aerosol-generating procedures are to be undertaken measurement among the community and health workers (Seto et al., 2013). 

At present, COVID-19 is causing secondary outbreaks across the globe. The effective control of this fatal outbreak is much dependent upon the isolation of infected individuals and the quarantine of suspected individuals. The current scenario indicates the need for high-level public health interventions such as intensive contact tracing as well as the implementation of rigorous vigilance, prevention, and control measures for reducing the transmission risks (Tang et al., 2020). However, the continued increase in the confirmed cases, along with the importation of matters to previously unaffected countries, suggests the possible failure of our preventive strategies. Hence, implementing strict public health measures will not be sufficient enough to control and eliminate the threat caused by COVID-19. Alternative approaches are required to prevent the spread of COVID-19. Even though several clinical trials are ongoing to identify and evaluate the therapeutic and preventive potential of several drugs and vaccines against SARS-CoV-2. However, more time will be required for making the final product available to the infected individuals. The development of specific antiviral drugs against COVID-19 will help to limit the morbidity and mortality while the vaccines and prophylactics will help to prevent the occurrence of new cases (Park et al., 2020).

## 13. Conclusion and prospects

In the last few decades, coronaviruses have represented a continuous pandemic threat to humans, and yet another outbreak caused by SARS-CoV-2 was firstly reported in Wuhan, China. The disease got spread across the country, and confirmed cases of SARS-CoV-2/COVID-19 had been published now in more than 80 countries and thus posed a “public health emergency of international concern.” This virus presently attained a high-risk category status by WHO. Community-based interventions such as school dismissals, event cancellations, social distancing, and the creation of staff plan to work remotely can help slow down the spread of COVID-19 as there are currently no vaccine and specific antiviral drugs available. Individuals can take daily preventive measures, such as frequent hand washing, staying at home when they are sick, and coughing and sneezing (CDC, 2020; Wu and McGoogan, 2020). Besides, it has been recommended to avoid a visit to live-animal markets. China has approved 10 detection kits used for COVID-19. The rapid serological diagnostic kit is being developed but not widely used (Report, 2020). Detection of SARS-CoV-2 from respiratory secretions by Quantitative Real-time RT-PCR (RT-qPCR) assay has routinely been used. In the context of the current situation in China, especially in Hubei province, for comprehensive management of the outbreak, a combination of RT-qPCR or NGS and clinical criteria may be more critical. 

Outside China, a dramatic increase of COVID-19 cases has been observed in South Korea, Iran, Italy, Japan, France, and Spain. There is a dire need for policies and efforts at national, regional, and international to stop further spreading of disease. Therefore, collaboration, cooperation, exchange of outbreak experience and response, strengthening of health facilities and disease surveillance, cross border monitoring, and an effective vaccine and treatment are needed. Furthermore, One Health should be of great importance. Future research studies and high investments are of significant concerns and have high stress to prevent the next epidemic and pandemic. Three areas need to be strengthened, a) improvement of wildlife trade market biosecurity b) identification and surveillance of high-risk pathogens among wildlife c) surveillance among people who have contact with wildlife or farm animals to avoid and prevent any future spillover of diseases from animals to humans (Daszak et al., 2020). 

## Acknowledgement

The authors acknowledge their respective institutes/universities. 
